# UAV Visual and Thermographic Power Line Detection Using Deep Learning

**DOI:** 10.3390/s24175678

**Published:** 2024-08-31

**Authors:** Tiago Santos, Tiago Cunha, André Dias, António Paulo Moreira, José Almeida

**Affiliations:** 1 INESCTEC—Institute for Systems and Computer Engineering, Technology and Science, Rua Dr. Roberto Frias, 4200-465 Porto, Portugal; andre.dias@inesctec.pt (A.D.); antonio.p.moreira@inesctec.pt (A.P.M.); jose.m.almeida@inesctec.pt (J.A.); 2 ISEP—School of Engineering, Polytechnic Institute of Porto, Rua Dr. António Bernardino de Almeida 431, 4200-072 Porto, Portugal; 1180922@isep.ipp.pt; 3 FEUP—Faculty of Engineering, University of Porto, Rua Dr. Roberto Frias, 4200-465 Porto, Portugal

**Keywords:** deep learning, UAV, power lines, inspection, thermographic images

## Abstract

Inspecting and maintaining power lines is essential for ensuring the safety, reliability, and efficiency of electrical infrastructure. This process involves regular assessment to identify hazards such as damaged wires, corrosion, or vegetation encroachment, followed by timely maintenance to prevent accidents and power outages. By conducting routine inspections and maintenance, utilities can comply with regulations, enhance operational efficiency, and extend the lifespan of power lines and equipment. Unmanned Aerial Vehicles (UAVs) can play a relevant role in this process by increasing efficiency through rapid coverage of large areas and access to difficult-to-reach locations, enhanced safety by minimizing risks to personnel in hazardous environments, and cost-effectiveness compared to traditional methods. UAVs equipped with sensors such as visual and thermographic cameras enable the accurate collection of high-resolution data, facilitating early detection of defects and other potential issues. To ensure the safety of the autonomous inspection process, UAVs must be capable of performing onboard processing, particularly for detection of power lines and obstacles. In this paper, we address the development of a deep learning approach with YOLOv8 for power line detection based on visual and thermographic images. The developed solution was validated with a UAV during a power line inspection mission, obtaining mAP@0.5 results of over 90.5% on visible images and over 96.9% on thermographic images.

## 1. Introduction

In the contemporary world, electricity has become one of the most essential commodities for human life. It plays a pivotal role in powering essential household appliances, illumination, and many currently available technologies. It is also crucial for retail, educational, and medical establishments, among others. In the face of ambitious global initiatives such as the commitment to achieve net-zero emissions by 2050 [1], challenges arise around reduction of emissions. Related to this, there is an evident surge in the adoption of renewable energy sources and the electrification of sectors such as transportation and heating. This transition translates into a foreseeable increase in the demand for electricity. In light of this trend, there is no doubt about the need for electricity distribution companies ro guarantee continuous power delivery without fail, as the alternative could lead to catastrophic consequences. Companies need to implement redundancy and backup strategies to avoid power outages. Redundancy can be achieved by using multiple power line routes to the same place; however, the population can reject the installation of new power lines [2]. Another solution is to take care of the already implemented network; for this, predictive maintenance is vital to ensuring timely preventive action.

The inspection of electrical assets is an important maintenance task in which periodic visual and thermal inspections are carried out in order to find failures in power components [3,4,5]. In the past, these inspections were carried out by human patrols [2,6,7,8] or manned aerial vehicles [9,10]; today, it is possible to use Multi-Rotor UAVs equipped with similar sensors that can get closer to the assets, taking advantage of their high maneuverability and capability to operate in harsh environments.

Unmanned Aerial Vehicle (UAV) application scenarios are increasing rapidly due to the current research interest in aerial robotics [11]. There are many different types of UAV configurations that present multiple structural designs, mirroring the vast number of UAV applications. Fixed-wing and multi-rotor UAVs provide a flexible approach for close inspection of structures, resulting in data with high resolution. Electrical power providers have great interest in these capacities [12,13,14,15,16], as they can reduce human risk and simultaneously collect data from different positions, angles, and distances with respect to electric assets. In this sense, it is critical to develop perception systems onboard the UAV that allow it to detect obstacles and electrical power lines during flight. In this paper, we intend to address this problem, focusing specifically on the development of an onboard perception system for a UAV that allows for tracking of electrical power lines. Equipping UAVs with the ability to perceive electric power lines will allow for future implementation of anomaly inspection strategies (i.e., corrosion, hot spots, etc.). Therefore, this paper contributes to ongoing initiatives to enhance the real-time perception capabilities of Unmanned Aerial Vehicles (UAVs) by equipping them with advanced capabilities for secure and resilient autonomous and semi-autonomous operation. This study focuses on using instance segmentation to detect power lines in images of visual and thermographic cameras that have been synchronously triggered to obtain the images at the same time.

The field of power line inspection has been a focal point for researchers, who are continually exploring innovative solutions to improve the accuracy, efficiency, and safety of these critical operations. Significant advancements have been made in remote sensing methods, as highlighted in the comprehensive review by Matikainen et al. [6].

The following literature review concentrates on key sensors, specifically visual and thermographic cameras, as well as LiDar in the context of power transmission line detection. The emphasis is predominantly on visual and thermographic cameras, aligning with the research findings presented in this study. Aerial images captured via UAV allow data on an object to be acquired in great detail due to UAVs’ close flight capability. Visual images are used for power line detection, while thermal images [17,18,19,20] are usually used for fault monitoring (i.e., hot spots) in power line components.

Traditional vision-based power line detection methods are divided into two significant steps, namely, segment detection/extraction and power line segment extraction. In segment extraction, methods used by algorithms include edge extraction, gray image thresholding [21,22,23], line detector masks, ratio line detection [24], the CVK method [25], canny edge detection [26,27], edge drawing [28], and Marr–Hildreth detection [29]. After segment extraction, the Hough transform is typically used as the core of these power line detection algorithms [21,23,25,26,29,30,31], although it has high computational requirements.

With the growth of the deep learning field in recent years, numerous works have sought to solve detection problems using deep learning techniques. Power line detection is one such problem, as predicted by Nguyen [32]. One of the first works to use deep learning for power line detection was presented by Varghese et al. [33], who retrained the last few layers in a pretrained CNN model for multi-class classification of power infrastructures and achieved a 70% F-score for power line detection. Adam Stambler et al. [34] proposed Deep Wire CNN to detect the lines in the image, followed by a triangulation technique to estimate the 3D location of the lines. To combat the limitations of traditional approaches, Nguyen et al. [35] introduced LS-Net, a fast single-shot line segment detector, and applied it to the power line detection problem. Although they used synthetic images, the results showed that the proposed method could outperform other approaches. Motivated by the potential of instance segmentation, Li et al. [36] proposed the CableNet algorithm based on fully convolutional networks, with two improvements to consider the specific appearance characteristics of transmission lines. First, the convolutional layers were configured to better represent continuous long and thin shapes; second, the output consisted of multidimensional feature maps (i.e., segmentation). This resulted in the assignment of a cable ID to each power line. An approach presented by Song et al. [37] involves tagging each power line; first, a Mask Region-based Convolutional Neural Network (R-CNN) algorithm is used for preliminary extraction, followed by a line-fitting algorithm that solves the problem of broken and mis-extracted power lines. Line fitting over the CNN result was used by Diniz et al. [38], with the images processed through a YOLOV4 network to determine the presence ir absence of power lines. The resulting bounding box is isolated and filtered, then the probabilistic Hough transform is used to estimate the lines. YOLOV3 was used by Son et al. [39] to detect power lines to ensure the safety of agricultural spraying drones. Given the difficulty of labeling power lines as objects, this approach based on the tiny-YOLOv3 network uses several continuous boxes of a specific size. The YOLO series of models started in 2015 [40] with the YOLOv1 model. It initially used a single convolutional neural network (CNN) to detect objects in an image; however, some two-stage models were more accurate at the time. Multiple models were developed over the following years in efforts to overcome various limitations and enhance performance [41]. YOLOv8 is the model most recently released by Ultralytics.

Choi et al. presented a work using thermographic and visible images for power line detection [42]. They compared the performance of an existing fast segmentation convolutional neural network (Fast-SCNN) with a modified version in which additional convolution layers and transposed convolution layers were inserted to improve the performance. An interesting conclusion was that using an image contrast enhancement algorithm such as CLAHE [43] can allow for a clearer view of the power lines, thereby improving the detection results. Motivated by the problem of the class imbalance in deep learning, Yang et al. [44] proposed a power line extraction network called (PLE-Net) based on an encoder–decoder framework. They used the U-net network as the baseline and optimized it using an attention and feature enhancement block. PLE-Net was tested with both visible and infrared images.

Damodaran et al. [45] proposed a pipeline that integrates various elements, preprocessing techniques, deep learning models, classification algorithms, and the Hough transform. The images first passed through the Canny edge detector for better segmentation, followed by Otsu thresholding. Deep learning models were subsequently used for feature extraction. RsurgeNet, which uses VGG16 convolution layers along with addition layers and multiplication layers, was created to extract more features; the AlexNet, Vgg16, and ResNet-50 frameworks were also tested for comparison with the results. Three different classification algorithms were tested to classify the features (AdaBoost, Light GBM, and XgBoost). Finally, the Hough transform was employed to extract the semantic information of power lines.

Multiple authors have highlighted the accuracy and speed advantages of YOLOv8, which allow its use in real-time applications [46,47,48]. The above literature review has covered several methods of detecting lines using visible and thermographic cameras; however, none of the works presented concern the issue of integration with a UAV in a real application scenario. All previous authors have focused on the perception component rather than its integration into an actual vehicle that has to perform the mission of tracking power lines in real time. Thus, the research work presented here seeks to address the following lines of inquiry:

- A processing architecture for a UAV that allows multi-sensory synchronization of acquired information, allowing the power line-following method to be implemented. The solution proposed in Section 2 can also be scaled in the future to integrate new sources of information such as LiDAR [49].
- A dataset of synchronized and georeferenced visible and thermographic images with labels.
- A processing pipeline for visible and thermographic images based on YOLOv8.
- A summary of the experimental results and discussion of topics to be addressed in future research.

Following this motivation, the paper outline is as follows. In Section 2, our multimodal power line detection approach is defined. Section 3 focuses on the processing pipeline implemented for power line detection. Section 4 presents the constructed dataset of power line images and describes the methodology used to train the neural networks. Section 5 shows the results obtained with the implemented approach. Finally, Section 6 draws some conclusions on the work carried out thus far and describes future work.

## 2. Multimodal Power Line Detection Approach

**Figure 1:**
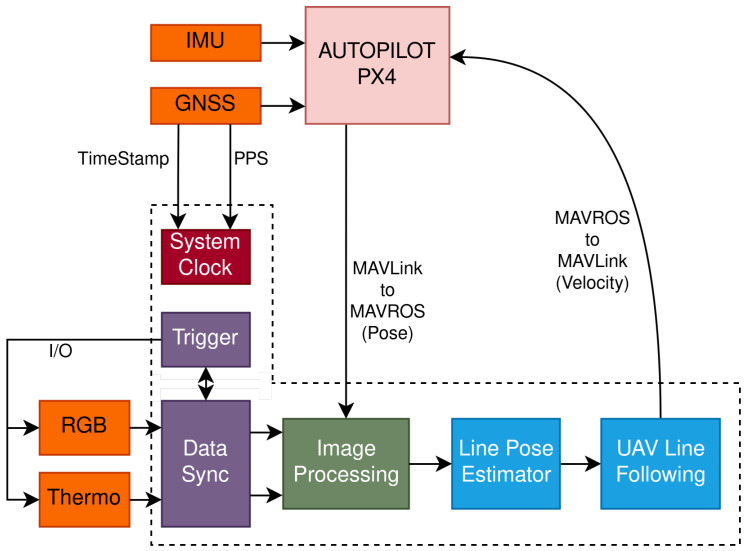
Overview of the software and hardware architecture with data flow.

Conducting power line inspections with UAVs entails a multifaceted approach that extends beyond deploying sensors such as visual or thermographic cameras. To equip a UAV with autonomous navigation capacities, integration of various other crucial technologies is required, including synchronization mechanisms, GPS for accurate positioning, and data processing systems. Figure 1 shows an architecture that mixes current work with future work.

The dashed box represents the companion computer that is connected to the autopilot. Autopilot data are mapped to the ROS environment by the MAVROS package, with MAVLink messages being exchanged between the two modules.

On the companion computer, the system clock is updated using the chrony application, a versatile implementation of the Network Time Protocol (NTP), which uses the GNSS timestamp and the precise pulse per second (PPS) signal to synchronise the system clock with the GNSS reference clock. This approach bases the system clock on a global reference, facilitating a multi-system integration if needed as well as a precise relation between the timestamps of the data.

The orange boxes in the figure represent the primary sensors present in the UAV, composed of the IMU and GNSS, which provide centimeter-level accuracy through the RTK technique and the visible and thermographic camera sensors. The two cameras have an I/O signal line triggered at the same time to capture an image, which is confirmed by data synchronization for correspondence between the trigger and the images. The green Image Processing box, representing the work presented here, takes the images and processes them to detect the power line. The blue boxes show future work, where the pose of the lines is estimated from the detections to serve as input for the line-following maneuver.

## 3. Implemented Image Processing Approach

**Figure 2:**
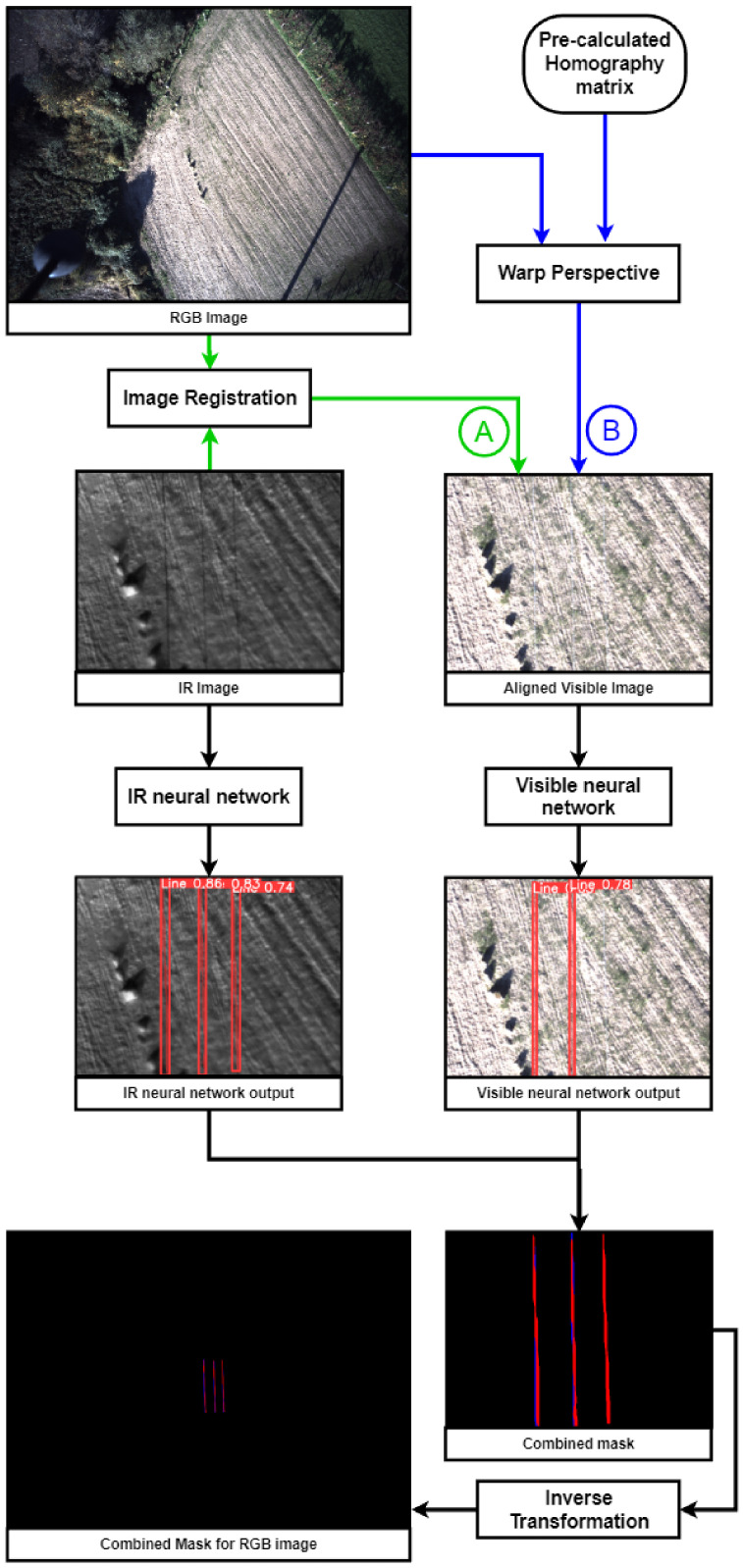
Processing pipeline.

The two cameras have different resolutions and FOVs; thus, an image registration technique is used to align and crop the visual image with the thermographic image. Each of these images is then passed to a corresponding neural network previously trained using that type of image. Finally, the results for each image are combined into a single output mask. Figure 2 details the approach used to address this problem.

The use of the homography matrix resolves the image registration problem. Although the homography matrix is typically used to align planes in an image, given the baseline of the cameras and the flight distance to the power lines, the visual result of the alignment is satisfactory, showing the potential of the solution.

Based on the YOLOv8 algorithm, one neural network is trained to detect power lines in thermographic images and another to detect power lines in visual images.

## 4. Dataset and Data Preparation

**Figure 3:**
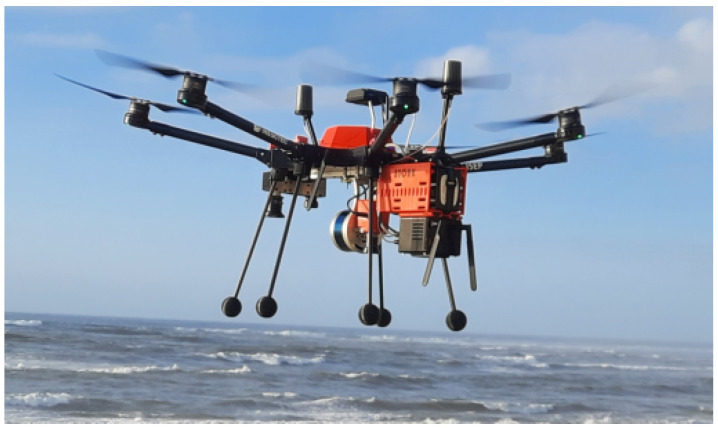
STORK UAV during an inspection mission.

The multirotor STORK UAV [50], shown in Figure 3, was used to carry out a flight over the power lines in the field test area. This UAV is used by the Institute of Systems and Computer Engineering Technology and Science (INESC TEC) in the development of several projects in the field of autonomous UAVs [49,50,51,52] due to its payload and adaptability that allow the vehicle to be customized for other application scenarios.

**Figure 4:**
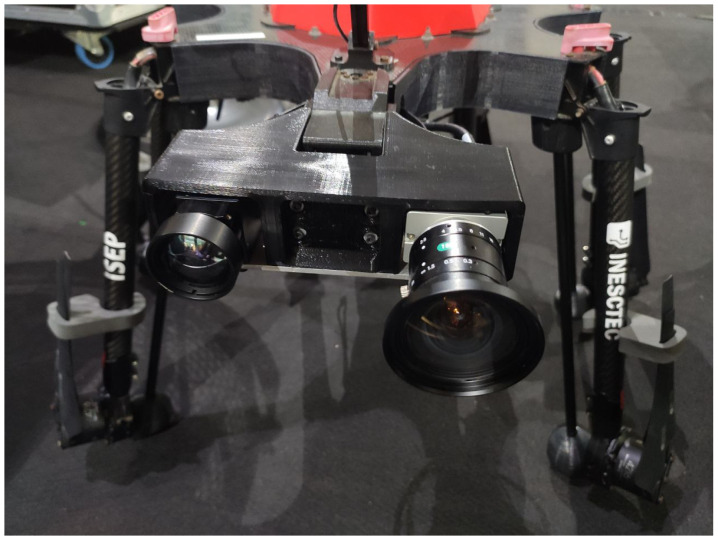
Onboard thermographic and visual camera on STORK UAV.

The UAV was equipped with two cameras, as shown in Figure 4, a Teledyne dalsa calibir DXM640 IR-GMZG-4104500 thermographic camera and a Genie Nano C4020 G3-GC11-C4020 visual camera. The thermographic camera has a 640 × 480 resolution with a field of view (FOV) of 24.2° × 18.4°, while the visual camera has a 4112 × 3008 resolution with a FOV of 83.0° × 66.0°. The flight was made over the power lines with the cameras pointing down. Both cameras were synchronized with a frame rate of 4 fps to correlate the visible and thermographic images.

**Figure 5:**
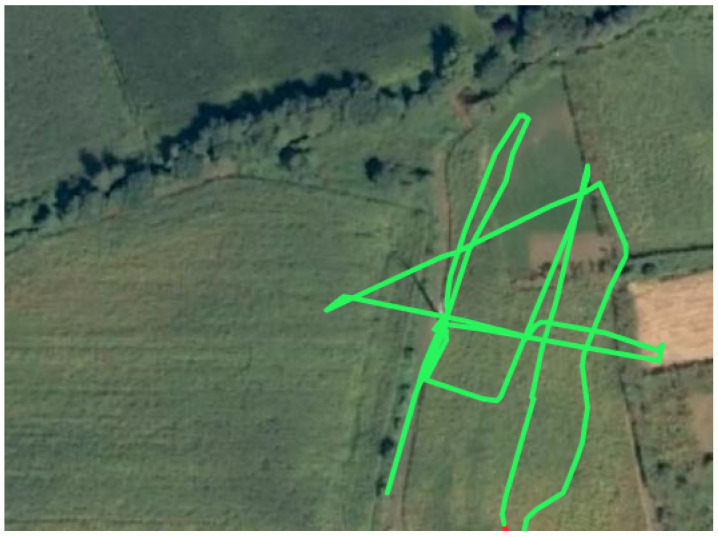
UAV trajectory (green line) during inspection of the power lines.

Two flights were conducted, one in the morning and another in the afternoon. A total of 3600 images were acquired, with 1800 captured by each camera. Figure 5 depicts the trajectory of a manual flight.

**Figure 6:**
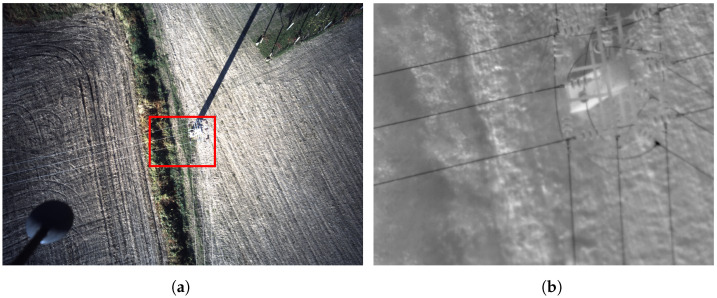
Tower with five intersecting power lines: (**a**) RGB image, with red square representing the placement of thermographic image; (**b**) thermographic image.

The location of the flight was chosen based on the presence of a central power tower with five intersections between different power lines corridors, as shown in Figure 6. In these two images, the difference in FOV is very noticeable. The red square on the RGB image represents the region that the thermographic camera captures.

The thermographic camera is capable of output with 14 bits per pixel. Adaptive contrast enhancement is implemented to output with 8 bits per pixel. The 14-bit image is analyzed to determine the minimum and maximum pixel value. The minimum pixel value is assigned to a value of 0 (black) and the maximum pixel value is assigned to 255. All the values in between are mapped between 0 and 255 to form the final grayscale image.

**Figure 7:**
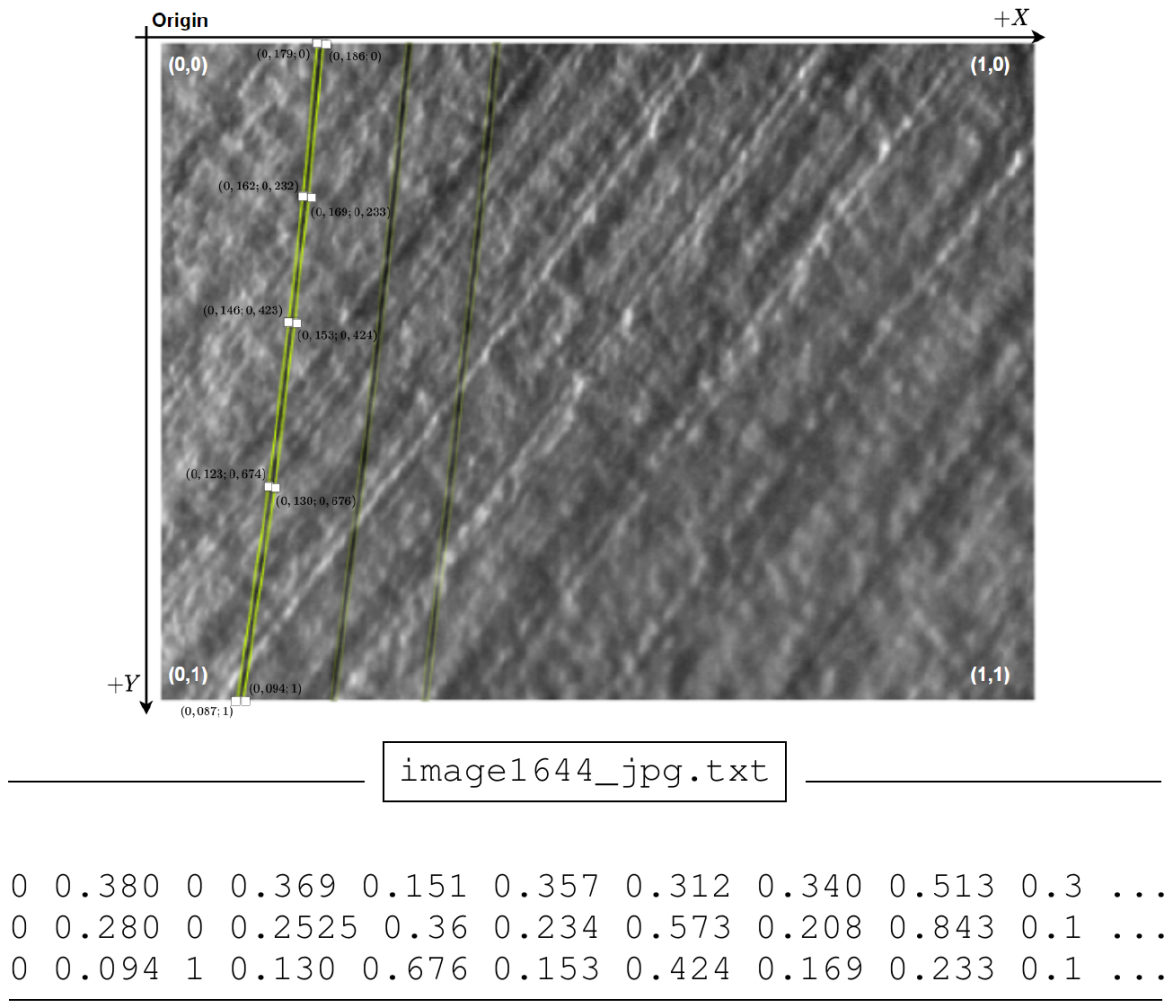
Segmentation points annotation example.

YOLOv8 uses the same format as the previous v5 iteration. For image segmentation, the annotations are different from those for object detection. Each image has a corresponding .txt file, with each line corresponding to each object segmentation. The line starts with the class ID, followed by multiple (*x*,*y*) normalized coordinates that describe the form of the segmentation, as shown in Figure 7.

The labeling process was performed in the Roboflow online application, which can augment data in the images to improve the generalization of the model and achieve greater performance on unseen images. In certain images, mainly the visible images, it can be challenging to correctly annotate the power lines due to the background. The dataset comprises 521 thermographic images and 515 visible images for training, with 50 of each type used for validation and 26 used for testing.

As previously stated, YOLOv8 has five pretrained scaled versions, with the *-seg* suffix used for image segmentation models. Table 1 shows the differences in performance between the five models. This work uses the YOLOv8n and YOLOv8s segmentation models.

## 5. Results and Discussion

YOLOv8n and YOLOv8s were used to train the models used to detect power lines. There are several key hyperparameters to consider that can affect the model accuracy, including batch size (which uses more memory, but can stabilize training), learning rate (weight update steps, which allows for precise adjustments yet slows down convergence), image size (all images are resized to this dimension), epochs (total number of full passes over the entire dataset), and patience (number of epochs without improvement in validation metrics, used for early stoppage of training).

The training phase was configured to run over 500 epochs, with the two models trained using the same default parameters except for the patience level, which was set as patience=150 for YOLOv8n and patience=50 for YOLOv8s. Decreasing the patience allows faster training, but can lead to worse performance.

Both YOLOv8s models were interrupted by the patience parameter, in epoch 229 for the RGB model and epoch 187 for the IR model. For YOLOv8n, the RGB model was interrupted in epoch 379, while the IR model used all 500 epochs. Despite the lower patience value used for YOLOv8n, it took more epochs for training compared to YOLOv8s. In the case of the IR model, the patience parameter was not used, and the model ran for the full 500 epochs.

**Figure 8:**
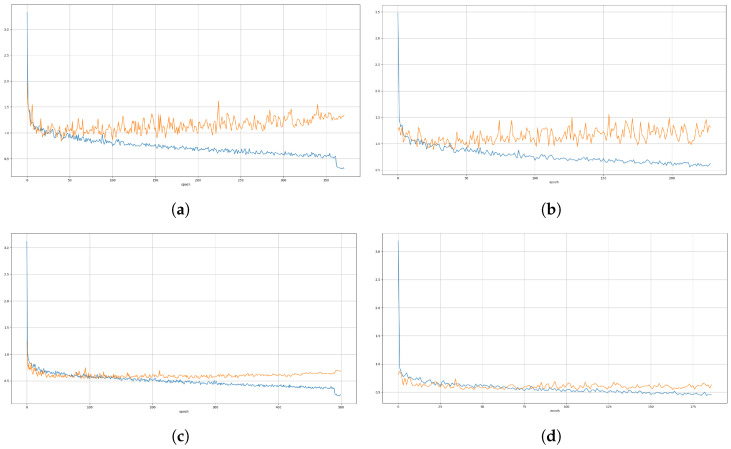
Model training loss (blue line) and validation loss (orange line): (**a**) YOLOV8n RGB model, (**b**) YOLOV8s RGB model, (**c**) YOLOV8n IR model, (**d**) YOLOV8s IR model.

**Figure 9:**
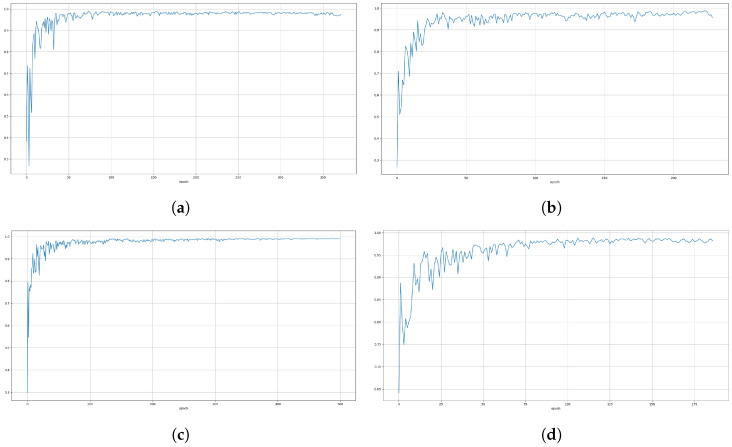
Model mean average precision (mAP@0.5): (**a**) YOLOV8n RGB model, (**b**) YOLOV8s RGB model, (**c**) YOLOV8n IR model, (**d**) YOLOV8s IR model.

For each model and camera, Figure 8 shows the training loss (blue) and validation loss (orange), while Figure 9 shows the mean average precision (mAP@0.5).

After training the models, the validation dataset was used to evaluate their performance. For this task, a fixed confidence threshold of 0.5 was selected for both types of images.

The inference time (milliseconds per image) is a critical aspect to consider, as it shows the processing time for each image. Table 2 shows the summary of the results, with the running times are compared to the previously trained models on an Intel i5-9300H CPU and Nvidia GeForce GTX 1650 GPU. The preprocessing step corresponds to the alignment between the images from the two cameras, while the postprocessing step refers to the combination of the two obtained masks.

As expected, the inference time with the GPU is considerably lower than with the CPU. Although it is expected that the final real-time processing solution onboard the UAV will require the use of a GPU, we present the results obtained with a CPU equivalent to the one currently equipped on the UAV STORK used to produce the dataset. In addition, the difference in performance based on the selected model is notable, with the *nano* version is approximately two times faster than the *small* model.

In the labeling phase, there were cases of small or thin lines that were hard for the human eye to see; naturally, these were not annotated in order to avoid misleading the training. However, the result was that the trained models could detect more lines than were annotated in the validation dataset. In the 50 images from each camera presented in the validation dataset, 131 lines were annotated in the visible images and 135 in the thermographic images.

Table 3 shows the results of instance segmentation on the validation dataset, referring to the metrics mAP@0.5 and mAP@0.5:0.95. From these results, the performance of the YOLOv8n instance seems to be better than that of the YOLOv8s instance, which was unexpected. From the training results in Figure 8 and Figure 9, it can be concluded that training of the YOLOv8n could be improved to achieve better results. However, given the detected instances and the masks produced for the images, the achieved results are satisfactory.

**Figure 10:**
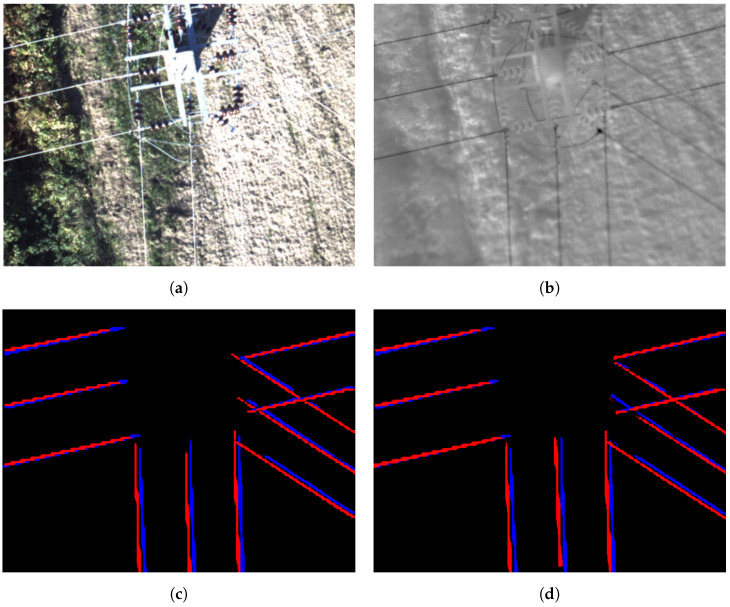
Example model predictions: (**a**) RGB image, (**b**) thermographic image, (**c**) YOLOV8n detection mask, (**d**) YOLOV8s detection mask.

Figure 10 shows an inference example for both models, with the blue lines representing the thermographic image detection and the red lines representing the visible image detection.

**Figure 11:**
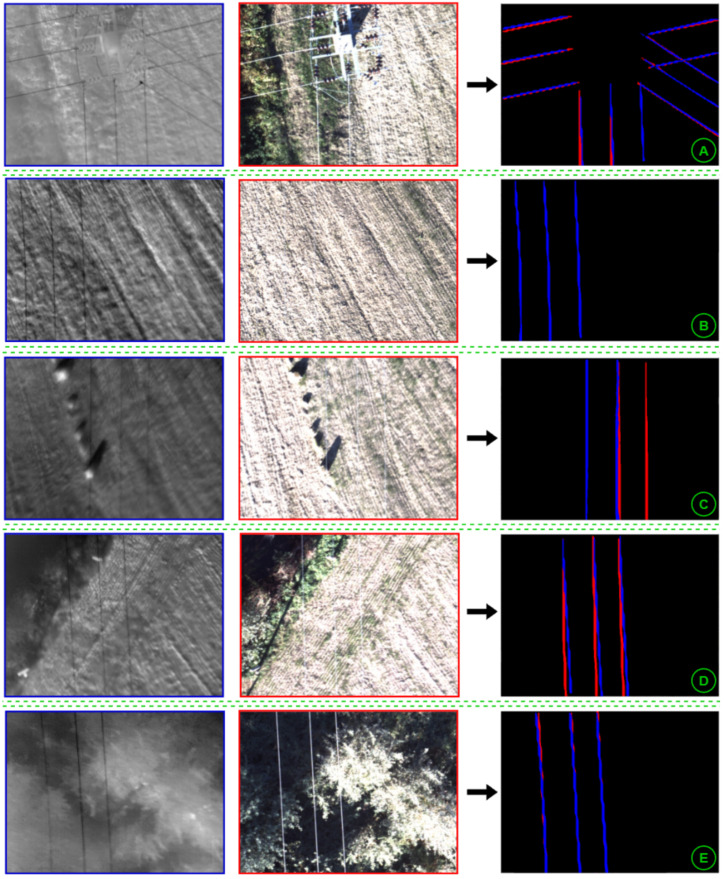
Detection examples on multiple images.

Figure 11 shows the obtained results in selected pairs of images. In section A (the tower with intersecting power lines), all lines were correctly detected. In section B, the RGB model was not capable of detection, however, the thermographic model correctly detected all three lines. In section C, the thermographic model failed while the RGB model was capable of detection. In section D, misalignment in the detections can be seen, most likely caused by the image registration technique. Finally, section E shows another successful detection.

## 6. Conclusions and Future Work

In this study, we have addressed the problem of power line detection by a UAV, proposing a solution based on YOLOv8 segmentation applied to visible and thermographic images synchronously acquired by the STORK UAV. Two flights were conducted in a specific area with five power line spans connected to a single power tower. Due to significant differences in FOV and size between the two image types, a preprocessing step was implemented in which the visual image is cropped and aligned with the thermographic image. YOLOv8n and YOLOV8s scaled versions were trained to detect power lines for each type of image. The experimental results show the efficacy of both models, with the YOLOv8n model achieving 98.4% mAP@50 on thermal images and 91.8% mAP@50 on visible images and the YOLOv8s model achieving 96.9% mAP@50 on thermal images and 90.5% mAP@50 on visible images. The YOLOv8n model performs better than the YOLOv8s model due to the suboptimal training of the YOLOv8s model. Even though a lower patience value was used, the IR model trained over all 500 epochs. Increasing the number of epochs and patience could lead to better results by both models. However, it is important to highlight that both models demonstrated the ability to segment power lines from both visible spectrum and thermographic images. This work has allowed us to validate AI techniques, in particular through the YOLO framework, in the context of line detection (segmentation) in visible and thermographic spectrum cameras and through the conceptualization of a processing architecture that in the future will allow processing onboard the UAV using edge computing. As this is an ongoing research topic, several weaknesses and challenges remain to be addressed. First, the image registration technique should be addressed in order to open up future possibilities involving instance segmentation, as in certain cases misalignment of the detections in the combined mask occurred during image registration. Second, the results revealed instances where detection failed in visible images while succeeding in thermographic images, and vice versa; a combination of the two sources of information could be studied to mitigate possible detection failures, and other sources of information such as LiDAR could be integrated into the proposed architecture. Third, at the time of this study YOLOv8 was the latest iteration of the model released by Ultralytics; however, this field is constantly evolving, with newer models such as YOLOv9 being released. The release of new models does not necessarily mean a clear improvement over the previous versions, as Jaykumaran concluded in his study comparing YOLOv8 and YOLOv9 [54]; nonetheless, emerging AI models such as RT-DERT [55] should be analyzed and tested to seek the best results and the possibility of executing them in real time.

## Figures and Tables

**Table 1:** YOLOv8 pretrained segment models [53].

Model	Size (Pixels)	mAP^box^ 50–95	mAP^mask^ 50–95	Speed CPU ONNX (ms)	Speed A100 TensorRT (ms)	Params (M)	FLOPs (B)
YOLOv8n-seg	640	36.7	30.5	96.1	1.21	3.4	12.6
YOLOv8s-seg	640	44.6	36.8	155.7	1.47	11.8	42.6
YOLOv8m-seg	640	49.9	40.8	317.0	2.18	27.3	110.2
YOLOv8l-seg	640	52.3	42.6	572.4	2.79	46.0	220.5
YOLOv8x-seg	640	53.4	43.4	712.1	4.02	71.8	344.1

**Table 2:** Runtime comparison of the models (ms/image) on an Intel i5 9300H CPU and Nvidia GeForce GTX 1650 GPU.

**Model**	**GPU**					
	**Thermal**			**Visible**		
	**Pre-Process (ms)**	**Inference (ms)**	**Post-Process (ms)**	**Pre-Process (ms)**	**Inference (ms)**	**Post-Process (ms)**
YOLOv8n	2.5	8.3	0.9	2.4	7.5	0.9
YOLOv8s	2.5	16.6	0.8	2.6	16.6	1.0
**Model**	**CPU**					
	**Thermal**			**Visible**		
	**Pre-process (ms)**	**Inference (ms)**	**Post-process (ms)**	**Pre-process (ms)**	**Inference (ms)**	**Post-process (ms)**
YOLOv8n	1.9	99.1	0.3	2.1	99.2	0.3
YOLOv8s	2.1	213.2	0.2	2.2	210.2	0.3

**Table 3:** Instance segmentation results on the validation dataset.

**Thermal Images**					
**Model**	**Detected** **Instances**	**Box**		**Mask**	
		**mAP@0.5** **(%)**	**mAP@[0.5:0.95]** **(%)**	**mAP@0.5** **(%)**	**mAP@[0.5:0.95]** **(%)**
YOLOv8n	141	98.4	93.0	98.4	65.2
YOLOv8s	141	96.9	88.9	96.9	62.3
**Visible Images**					
**Model**	**Detected** **Instances**	**Box**		**Mask**	
		**mAP@0.5** **(%)**	**mAP@[0.5:0.95]** **(%)**	**mAP@0.5** **(%)**	**mAP@[0.5:0.95]** **(%)**
YOLOv8n	135	97.0	86.7	91.8	47.3
YOLOv8s	135	95.7	85.5	90.5	46.2

## Data Availability

Data are contained within the article.
